# On-pump beating heart coronary surgery for high risk patients requiring emergency multiple coronary artery bypass grafting

**DOI:** 10.1186/1749-8090-3-38

**Published:** 2008-07-02

**Authors:** Enrico Ferrari, Nicolas Stalder, Ludwig K von Segesser

**Affiliations:** 1 Department of Cardiovascular Surgery, University Hospital of Lausanne, Lausanne, Switzerland

## Abstract

**Background:**

Cardiopulmonary bypass (CPB) with aortic cross-clamping and cardioplegic arrest remains the method of choice for patients requiring standard myocardial revascularization. Therefore, very high-risk patients presenting with acute coronary syndrome, unstable angina, onset of cardiac decompensation and requiring emergency multiple myocardial revascularization, can have a poor outcome. The on-pump beating heart technique can reduce the mortality and the morbidity in such a selected group of patients and this report describes our clinical experience.

**Methods:**

Out of 290 patients operated for CABG from January 2005 to January 2006, 25 (8.6%) selected high-risk patients suffering from life threatening coronary syndrome (mean age 69 ± 7 years) and requiring emergency multiple myocardial revascularization, underwent on-pump beating heart surgery. The mean pre-operative left ventricle ejection fraction (LVEF) was 27 ± 8%. The majority of them (88%) suffered of tri-vessel coronary disease and 6 (24%) had a left main stump disease. Nine patients (35%) were on severe cardiac failure and seven among them (28%) received a pre-operative intra-aortic balloon pump. The pre-operative EuroScore rate was equal or above 8 in 18 patients (73%).

**Results:**

All patients underwent on-pump-beating heart coronary revascularization. The mean number of graft/patient was 2.9 ± 0.6 and the internal mammary artery was used in 23 patients (92%). The mean CPB time was 84 ± 19 minutes. Two patients died during the recovery stay in the intensive care unit, and there were no postoperative myocardial infarctions between the survivors. Eight patients suffered of transitorily renal failure and 1 patient developed a sternal wound infection. The mean hospital stay was 12 ± 7 days. The follow-up was complete for all 23 patients survived at surgery and the mean follow-up time was 14 ± 5 months. One patient died during the follow-up for cardiac arrest and 2 patients required an implantable cardiac defibrillator. One year after surgery they all had a standard trans-thoracic echocardiogram showing a mean LVEF rate of 36 ± 11.8%.

**Conclusion:**

Standard on-pump arrested heart coronary surgery has higher mortality and morbidity in emergencies. The on-pump beating heart myocardial revascularization seems to be a valid alternative for the restricted and selected cohort of patients suffering from life threatening coronary syndrome and requiring multiple emergency CABG.

## Introduction

Use of cardiopulmonary bypass (CPB) with aortic cross-clamping and cardioplegic arrest remains the method of choice for patients requiring standard myocardial revascularization. This technique, which is used routinely worldwide to perform standard coronary artery bypass grafting (CABG), is still linked to several side-effects mostly due to the use of aortic cross-clamping, cardioplegic heart arrest and CPB, especially in emergency cases. During the last twenty years many efforts have been undertaken to reduce the incidence of major intraoperative and postoperative complications related to the procedure. In particular, shorter CPB circuits and newer cardioplegic arrests have been developed coupled with the beating-heart coronary surgery and the so-called "no touch technique" for vessel manipulation.

Despite low-risk patients requiring standard CABG having a poor risk to develop intraoperative and postoperative complications from the use of CPB and cardioplegic arrest, the subgroup of high-risk patients suffering from acute coronary syndrome with unstable angina and severe cardiac failure is extremely sensitive to emergency surgery: clinical and experimental trials present in literature confirm that use of CPB and cardioplegic arrest are strictly related with a multitude of pathogenic mechanisms responsible for the higher intraoperative and postoperative risk [1-3], but, on the other hand, patients suffering from unstable angina and undergoing emergency CABG are expected to derive the greatest benefits from multiple myocardial revascularization. In terms of left ventricular ejection fraction (LVEF), for example, an improvement will be expected if the ischemic and the hibernate myocardium could be restored after surgery and, moreover, some trials have already demonstrated that there are significant survival benefits for patients undergoing CABG and suffering from acute left ventricular dysfunction (preoperative LVEF<30%) due to extensive coronary disease [4,5].

To avoid the use of CPB, aortic cross-clamping and cardioplegic arrest, the off-pump coronary artery bypass grafting (OPCAB) technique was developed in the nineties with the specific purpose of reducing the mortality and the morbidity in high-risk and low-risk patients [6-8]. Unfortunately, during the extensive surgical manipulation and heart displacement necessary to perform multiple distal anastomoses, the OPCAB technique can cause episodes of transitory hemodynamic instability that could lead to secondary critical low coronary artery diastolic blood flow followed by severe complications or death.

The on-pump beating heart coronary surgery represents a merge of standard on-pump surgery and OPCAB technique. The absence of cardioplegic arrest coupled with the hemodynamic stability guaranteed during extensive heart manipulation, are the biggest benefits coming from this technique, especially in cases of unstable high risk patients. In the present study we describe our clinical experience with the on-pump beating heart coronary surgery for emergency multiple myocardial revascularization.

## Methods

From January 2005 to January 2006, out of 290 consecutive patients operated for isolated CABG in our department, 25 selected high-risk patients presenting with onset of acute coronary syndrome, unstable angina, signs of myocardial infarction and/or severe myocardial dysfunction, and not suitable for primary angioplasty or thrombolysis, were operated for emergency multiple myocardial revascularization using the on-pump beating heart coronary surgery technique. The major selection criteria were the absence of previous cardiac operation and the surgical myocardial revascularization as the lone suitable treatment for the life threatening coronary syndrome and/or initial myocardial infarction. The preoperative patients' characteristics are listed in Table 1. The mean age was 69 ± 7 years and 18 patients were male. The atherosclerosis risk factors distribution was the following: 65% active smokers, 56% suffering from systemic hypertension and 70% under treatment for hypercholesterolemia. A positive anamnesis of previous acute myocardial infarction was given in 13 cases (53%) and 9 patients (35%) were hospitalized for acute myocardial infarction during the week before surgery. Among them, nine patients (35%) developed signs of severe low cardiac output during or immediately after the cardiac angiogram, seven patients required a pre-operative intra-aortic balloon pump (IABP) implantation in the catheterization laboratory, and an emergency intubation with mechanical ventilation was necessary five times. All patients underwent a preoperative cardiac assessment with a chest X-ray, an EKG, a standard coronary angiogram and a trans-thoracic echocardiogram. Echocardiografically, the mean left ventricle ejection fraction (LVEF) rate was 27 ± 8%. Onsets of mitral valve dysfunction or regurgitation with left ventricular dilatation and/or focal dysfunction were important criteria for emergency coronary revascularization and pre-operative intra-aortic balloon pump positioning if other therapies were contraindicated (i.e. thrombolysis and primary angioplasty). Patients presenting with severe chest pain and/or low cardiac output were immediately treated with anti-platelet drugs, intra-venous heparin or nitroglycerin infusion, and/or inotropic drugs (Dobutamine, Noradrenaline) depending on the hemodynamic stability. The coronary angiogram showed that twenty two patients (88%) suffered from severe tri-vessel coronary artery disease and the left main stump disease was diagnosed 6 times.

**Table 1:** Baseline patient profile*

No. of Patients	25
Age (years)	69 ± 7 (range 57–79)
Gender (M/F)	18/7
CCS angina class	
I	0 (0%)
II	4 (16%)
III	13 (53%)
IV	8 (31%)
LVEF (%)	27 ± 8
Hypertension	14 (56%)
Smoke	16 (65%)
Hypercholesterolemia	17 (70%)
Diabetes mellitus (I&II)	4 (16%)
Peripheral vascular disease	9 (35%)
Prior myocardial infarction	13 (53%)
Myocardial infarction < 7 days	9 (35%)
Urgency	2 (8%)
Emergency	23 (92%)
Preoperative IABP	7 (28%)
Preoperative mechanical ventilation	5 (20%)
Severe low cardiac output	9 (35%)
Left main stump disease	6 (24%)
3 – Vessel disease	22 (88%)

* Data are presented as mean ± SD or N (%)

CCS: Canadian Cardiovascular Society Angina Class; New York Heart Association; LVEF: Left Ventricular Ejection Fraction; MI: Myocardial Infarction; IABP: Intra-Aortic Balloon Pump.

The pre-operative EuroScore [9] rate was equal or above 8 in 18 patients (73%) (Table 2) and all patients who survived surgery were followed up with an echocardiogram and a complete clinical examination one year after the surgery. The follow-up was 100% complete.

**Table 2:** Preoperative risk profile according to the Euroscore.

Preoperative Euroscore	Number of Patients, n (%)
6	3 (11%)
7	4 (16%)
8	5 (20%)
> 8	13 (53%)

### Surgical technique

Patients were prepared for surgery following the conventional guidelines for CABG and a trans-esophageal echocardiogram was routinely performed intraoperatively. Through a median sternotomy, all patients were cannulated in the standard way. On the beating heart, normo-thermia and full cardiopulmonary bypass (CPB), the grafts were distally anastomized to the coronary arteries using the CTS Axius Guidant Stabilizer system (Guidant Corporation, Santa Clara, CA, USA), beginning from the left mammary artery on the left anterior descending coronary artery. Intra-coronary shunts by Medtronic (Medtronic Inc. Minneapolis, MN, USA) were used when needed. The venous proximal anastomosis was performed immediately after each distal anastomosis directly to the ascending aorta using an aortic side-clamp and a cardio punch.

## Results

All 25 high-risk patients were operated for emergency multiple myocardial revascularization using the on-pump-beating heart technique. In our group there were no conversions to cardioplegic arrest and aortic cross clamping. The mean number of graft/patient was 2.9 ± 0.6 and the left internal mammary artery (LIMA) was used 23 times (92%). In two cases the mammary artery was not used because the time spent to harvest the mammary artery would have endangered the patient's hemodynamic stability, and in one case both mammary arteries were prepared due to a previous bilateral safenectomy. The left anterior descending coronary artery (LAD) was revascularized in all 25 patients (100%), one or more marginal branches from the circumflex coronary artery were grafted in 20 patients (82%) and a branch from the right coronary artery was revascularized in 22 cases (88%). The myocardial revascularization was complete for 23 patients (92%). The mean CPB time was 84 ± 19 minutes and the mean total operative time was 188 ± 36 minutes (intraoperative data are listed in Table 3).

**Table 3:** Intraoperative data*

No. of Patients	25
No. of graft/patient	2.9 ± 0.6
Use of LIMA	22 (88%)
Use of LIMA + RIMA	1 (4%)
Use of veins	25 (100%)
Target Coronary Arteries:	
Left anterior descending artery	25 (100%)
Diagonal branches	3 (12%)
Marginal branches or Circumflex	20 (82%)
Right coronary artery	22 (88%)
Complete revascularization	23 (92%)
Cardiopulmonary bypass time (min)	84 ± 19
Operative time (min)	188 ± 36

* Data are presented as mean ± SD or N (%)

LIMA: Left Internal Mammary Artery; RIMA: Right Internal Mammary Artery.

Two patients (8%) died during recovery in the Intensive Care Unit: the first one died following major and irreversible ventricular fibrillation one day after the procedure and the second patient died following mediastinitis and sepsis two weeks after the operation. Among the 23 survivors, no one developed a postoperative acute myocardial infarction and the mean CK peak rate was 1060 ± 1094 U/l, in line with the preoperative diagnosis of severe coronary syndrome and acute myocardial infarction. The intensive use of heparin, aspirin, clopidogrel and IIb/IIIa receptors' inhibitors before surgery, can explain a mean postoperative bleeding of 1340 ± 903 mL. Although, the surgical bleeding was diffuse and continuous, each patient's hemodynamic stability was never compromised, they all received fresh plasma and platelets if needed, and there were no re-thoracotomies for bleeding or for any other reason in our series. A transitory postoperative renal failure was also diagnosed in eight patients during recovery in the ICU and this was probably due to preoperative low cardiac output syndrome. The mean Intensive Care Unit stay was 4.4 ± 6.4 days and the mean global hospital stay was 12 ± 6.7 days (see Table 4). Before being discharged from the hospital, the 23 patients having survived surgery underwent a trans-thoracic echocardiogram showing a mean LVEF of 34.4 ± 8.5%.

**Table 4:** Postoperative results*

No. of patients	25
Hospital mortality	2 (8%)
Ventilation time (hours)	26 ± 37 (range 7–168)
Intraoperative IABP	3 (11%)
Total bleeding (mL)	1340 ± 903
Re-exploration for bleeding	0
Myocardial infarction	0
CK-MB peak (U/l)	1060 ± 1094
Postoperative LVEF (%)	34.4 ± 8.5
Low cardiac output	2 (8%)
Transitory acute renal failure	8 (32%)
Sternal infection	1 (4%)
Intensive care unit stay (days)	4.4 ± 6.4 (range 1–21)
Hospital stay (days)	12 ± 6.7 (range 8–34)

* Data are presented as mean ± SD or N (%)

IABP: Intra-Aortic Balloon Pump; CK-MB: Creatine Kinase-MB; LVEF: Left Ventricular Ejection Fraction.

Patients were followed-up for almost one year after surgery (mean follow-up time 14 ± 5 months): they all had a standard trans-thoracic echocardiogram and a clinical examination. Excluding one patient who died following cardiac arrest four months after the operation, all 21 survivors have an acceptable quality of life with a mean LVEF of 36 ± 11.8%. There were no cardiac re-operations, major neurological events or acute myocardial infarctions, although two patients required an implantable cardiac defibrillator (ICD) to prevent severe electrical dysfunctions originating from their ischemic cardiomyopathy. Follow-up details are listed in Table 5.

**Table 5:** Follow-up results*

Mean follow-up time (months)	14 ± 5
No. of patients	23
Internal cardiac defibrillator	2
Cardiac death	1
LVEF (%)	36 ± 11.8

* Data are presented as mean ± SD or N (%)

LVEF: Left Ventricular Ejection Fraction.

## Discussion

The optimal treatment for patients presenting with unstable angina, acute coronary syndrome, onset of myocardial infarction or severe left ventricular dysfunction and carrying a diffuse multi-vessel coronary artery disease is still controversial. Primary coronary angioplasty and systemic thrombolysis have been identified as fast and efficient treatments in case of severe and irreversible acute coronary syndrome but they can also be contraindicated, depending on specific concomitant factors. In particular, patients with severe multivessel coronary artery disease or main stump disease, presenting comorbidities that contraindicate the thrombolysis, or showing signs of acute and severe left ventricular dysfunction with low cardiac output requiring urgent mechanical circulatory support, can derive big benefits from emergency on-pump multiple myocardial revascularization. Nevertheless, the standard surgical technique, with cardioplegic arrest and cardiopulmonary bypass, may not be the ideal solution in this cohort of very high-risk and unstable patients: in particular, cardioplegic arrest and aortic cross clamping have been isolated as independent surgical risk factors for high-risk patients suffering from acute coronary syndrome and severe cardiac dysfunction, while the avoidance of cardiopulmonary bypass does not confer significant clinical advantages, as suggested by recent reports [10,11]. In particular, the report from Légaré et Al [11], in which two groups of patients undergoing CABG with CPB or on the beating heart are compared, does not demonstrate any difference between the two groups, with regards to postoperative mortality, morbidity and hospital stay length. Following those findings and in order to guarantee the best surgical results in this restricted group of patients, the beating heart technique for emergency CABG can be supported by the use of appropriate technical supports, like intra-aortic balloon pump, heart stabilizers, intra-coronary shunts and complete CPB [2-12]. In other words, in order to achieve a maximum long-term benefit while minimizing short-term risks, further surgical strategies have been recently explored and the on-pump beating heart coronary surgery has been reported as an acceptable trade-off between the conventional CABG with cardioplegic arrest and the OPCABG in selected unstable high-risk patients and in emergencies [13-15]. In our report we present the one year follow-up after multiple myocardial revascularizations under on-pump beating heart technique, in a series of 25 consecutive very high risk patients operated in emergency, out of 290 patients operated in the same period for standard CABG. In particular, our patients were not suitable for alternative non-surgical treatments, they were operated in the shortest delay, and they were preoperatively treated with IABP and/or high doses of inotropic drugs in order to achieve a certain degree of hemodynamic stability, when needed. The biggest benefits deriving from the on-pump beating heart technique were the reduction of the hemodynamic instability caused by surgical manipulations, the absence of global myocardial ischemia during aortic cross-clamping time and the absence of reperfusion after cardioplegic arrest. Despite some reports comparing the on-pump beating heart technique versus the standard CABG having already been published in literature [13-16], only a few of them have focused their attention to the selected cohort of emergency high-risk coronary patients [16,17]. In their report, for example, Edgerton et Al [17] described a series of 364 cases operated under on-pump beating heart technique and, among them, only 15 (4.1%) were classified as emergencies. In our series (25 patients), 92% of patients were operated in an emergency and, in 9 cases, there were clinical signs of severe life threatening low cardiac output. The Euroscore rate was equal or superior to eight 18 times. As expected in such a very high-risk group of patients, they often developed postoperative transitory acute renal failure (32%) or low cardiac output (8%) and two from 25 died during recovery in the intensive care unit (mortality rate 8%). Moreover, the patients' postoperative bleeding rate was higher than expected in a normal CABG group and this fact can be easily explained by the extensive use of high doses of heparin and anti-platelet drugs given preoperatively. The mean intensive care unit stay was also longer (4.4 ± 6.4 days) than expected in a standard CABG group and all of these findings are in line with comparable data already present in literature. Despite the fact that our cohort of patients requiring emergency on-pump beating heart surgery for CABG is a small group and that the results cannot be evaluated in the way they would have been if coming from a larger cohort of patients, we strictly believe that focused reports are still necessary to identify the best surgical approach in this selected diseased population. In our experience, the on-pump beating heart surgery in emergencies was used as the last way to save people from life-threatening symptoms and, to us, this treatment can guarantee acceptable results. The one year follow-up showed that the mean patients' left ventricle ejection fraction rate increased from 26 ± 8% (preoperative data including patients with IABP and high doses of inotropic drugs) to 36 ± 11.8% which is a good result and let patients live acceptable lives. Unfortunately, according to the presence of some degree of ischemic cardiac myopathy in patients who survived surgery, one patient died of irreversible ventricular fibrillation and two patients required an implantable cardiac defibrillator.

In conclusion, one of the main problems in patients undergoing emergency CABG remains the myocardial protection and the side effects coming from the transitory myocardial ischemia during arrested heart surgery (possibly due to the myocardial edema) [18,19]. Theoretically, off-pump beating heart surgery supported by inotropic drugs and an aortic balloon pump can be a suitable solution for high-risk emergency CABG despite the fact that in cases of cardiogenic shock the extensive mobilization and manipulation of the heart can lead to severe hemodynamic instability. In conclusion, although further reports and randomized clinical trials are necessary to compare results coming from different surgical strategies undertaken to treat such a subgroup of high-risk patients, we strongly believe that, following reported data and looking closely to our surgical activity in this field, the on-pump beating heart CABG surgery, when not strictly contraindicated (i.e. calcified aorta), can lead to acceptable short and mid-term results and remains an attractive alternative to conventional myocardial revascularization and off-pump beating heart surgery in emergency cases.

### Study limitations

This clinical study is a non randomized retrospective study followed by a one year follow-up. There is no control group because, during the observational period, all patients suffering from acute coronary syndrome followed by cardiac failure and referred to our department, were immediately sent for surgery and operated under on-pump beating heart technique. Moreover, the cohort of patients involved in our study represents a small and selected group of high-risk patients operated for CABG during the observational period (the 8.6% of all CABG cases). Although a bigger amount of patients would be mandatory to guarantee more statistically significant results coming from this surgical technique, we believe that our study can already show interesting results in terms of short-term and mid-term follow-up.

## Authors' contributions

EF has developed the entire project, he collected and analyzed clinical and surgical data and he wrote the article, NS contributed to collect data, in particular during the follow-up, and he contributed in achieving and analyzing data, LKvS supervised the project.
